# Histone Methylation Participates in Gene Expression Control during the Early Development of the Pacific Oyster *Crassostrea gigas*

**DOI:** 10.3390/genes10090695

**Published:** 2019-09-10

**Authors:** Alexandre Fellous, Lorane Le Franc, Aude Jouaux, Didier Goux, Pascal Favrel, Guillaume Rivière

**Affiliations:** 1 Unité de Formation et de Recherches (UFR) des sciences, Université de Caen Normandie, 14032 Caen CEDEX, France; 2 Biologie des Organismes et Ecosystèmes Aquatiques (BOREA), FRE2030, Muséum National d’Histoire Naturelle (MNHN), Centre National de la Recherche Scientifique (CNRS), Institut de Recherche et Développement (IRD), Sorbonne Université (SU), Université de Caen Normandie (UCN), Université des Antilles (UA), 75231 Paris CEDEX, France; 3 Coastal Ecology Section, Alfred Wegener Institute Helmholtz Centre for Polar and Marine Research, Wadden Sea Station Sylt, 25992 List, Germany; 4 Centre de Microscopie Appliquée à la Biologie, SF 4206 Interaction Cellule-Organisme-Environnement (ICORE), Université de Caen Normandie, Esplanade de la paix, 14032 Caen CEDEX, France

**Keywords:** epigenetics, histone modifications, methylstat, embryos, mollusk, H3K4, H3K9, H3K27, H3K36

## Abstract

Histone methylation patterns are important epigenetic regulators of mammalian development, notably through stem cell identity maintenance by chromatin remodeling and transcriptional control of pluripotency genes. But, the implications of histone marks are poorly understood in distant groups outside vertebrates and ecdysozoan models. However, the development of the Pacific oyster *Crassostrea gigas* is under the strong epigenetic influence of DNA methylation, and *Jumonji* histone-demethylase orthologues are highly expressed during *C*. *gigas* early life. This suggests a physiological relevance of histone methylation regulation in oyster development, raising the question of functional conservation of this epigenetic pathway in lophotrochozoan. Quantification of histone methylation using fluorescent ELISAs during oyster early life indicated significant variations in monomethyl histone H3 lysine 4 (H3K4me), an overall decrease in H3K9 mono- and tri-methylations, and in H3K36 methylations, respectively, whereas no significant modification could be detected in H3K27 methylation. Early in vivo treatment with the JmjC-specific inhibitor Methylstat induced hypermethylation of all the examined histone H3 lysines and developmental alterations as revealed by scanning electronic microscopy. Using microarrays, we identified 376 genes that were differentially expressed under methylstat treatment, which expression patterns could discriminate between samples as indicated by principal component analysis. Furthermore, Gene Ontology revealed that these genes were related to processes potentially important for embryonic stages such as binding, cell differentiation and development. These results suggest an important physiological significance of histone methylation in the oyster embryonic and larval life, providing, to our knowledge, the first insights into epigenetic regulation by histone methylation in lophotrochozoan development.

## 1. Introduction

Methylation of histone lysine residues is a common post-translational modification that impacts different cellular processes such as the regulation of transcription, nucleosome architecture, cell cycle and genome stability [1]. Lysines at the N-terminal end of the histones H3 and H4 are the most studied and are often associated with the compaction of chromatin and its transcriptional status [2]. Each lysine residue may be mono (me1), di (me2) or tri-methylated (me3), highlighting a high versatility of this epigenetic mark and a high variability in the chromatin landscapes regarding methylation, reflective of its very precise tuning potential of the nucleosome structure. Indeed, the heterochromatin has a compact structure associated with silent transcription and presents methylated forms of Histone 3 lysine 9 (H3K9), H3K27, and H4K20. Methylated H3K4, H3K36 and H3K79 residues [2] lie within the relaxed euchromatin and are associated with active transcription, although complex relationships exist between transcriptional activity and precise H3 lysine methylation patterns and combinations. For example, in eukaryotes, the transcription start sites of expressed genes are generally surrounded by H3K4me3, indicating a role in transcription initiation and early elongation for this epigenetic mark [3]. However, transcriptional outcomes of histone methylation depend on several parameters including number of methyl groups, residue bearing methylation, gene and cell types, chromatin context, and species examined [3]. Indeed, methylation of H3K9 has different outcomes depending on its localization within genes (regulating or coding regions) [2]. Physiologically, histone methylation patterns are widely implicated in the maintenance of the stem identity of cells and their differentiation. Indeed, the methylation of H3K4 forbids the local recruitment of DNMT3L during implantation in mammals and therefore controls selected gene expression in the early embryo through DNA methylation [4]. In a similar fashion, redifferentiation implies H3K4 demethylation by a JmjC histone demethylase [5], which participate in the transcriptional control of Oct3/4 and Nanog, illustrating the regulation of pluripotency genes expression by histone methylation [6,7]. Further illustrating the role of histone methylation in development, the methylation of H3K36 delimits body regions of active genes [8], while methyl marks on H3K27 are generally associated with transcriptional repression via Polycomb group (PcG) proteins [9]. Therefore, histone methylation patterns and their regulating factors are of paramount interest in a developmental context.

Among them, Jumonji-C domain containing proteins (JmjC) are a widely conserved family of histone demethylases, which can specifically remove methyl groups from lysines through a hydroxylase activity. These enzymes present a great diversity of domain architectures, which gives the JmjC specificity towards peculiar histone residues and/or number of methyl groups, as well as the ability to interact directly with DNA and/or other proteins [10,11,12]. Histone methylation in the development of model organism becomes increasingly understood [13]. However, despite such information being critical for our understanding of the evolution of epigenetic regulation, whether histones are dynamically methylated and whether this epigenetic pathway is of biological relevance remains poorly described in mollusks and more generally in the lophotrochozoans [14,15,16].

The pacific oyster *Crassostrea gigas* (Thunberg, 1793) (i.e., *Magallana gigas*) is a bivalve mollusk belonging to the lophotrochozoa, a distant evolutionary group that remains epigenetically poorly documented when compared to ecdysozoans (such as insects [17]), trematodes [18], nematodes [19] and vertebrates [13]. *Crassostrea gigas* undergoes an indirect development and adults are successive hermaphrodite animals, which live in the highly changing and stressful intertidal zone. Thus, throughout his life, the oyster undergoes many morpho-physiological changes, such as embryogenesis, metamorphosis, and annual gonad renewal. These changes rely on the proliferation and/or (re)differentiation of stem cells, that are supported by transient specific transcriptomes stabilized by the stochastic expression of developmental genes [20,21]. Because epigenetic mechanisms can establish and maintain such transcriptomes [22], they were hypothesized to be of physiological relevance in a developmental context. Consistently DNA methylation has been found important for the oyster embryogenesis [23,24,25,26,27,28]. In addition, our group recently demonstrated the presence of the JmjC histone demethylases family with strong and regulated mRNA levels during early life [29] together with an influence of temperature on histone methylation and *Jumonji* mRNA expression in embryos [15], suggesting a potential conservation of histone methylation and its functional outcomes in development. 

In order to gain more insights into this issue, we quantified histone methylation during oyster early life using fluorescent ELISAs and examined the effect of histone demethylation using methylstat, a specific inhibitor of JmjC enzymes, in vivo. In addition, to shed light on the possible biological roles of histone methylation during oyster development, we led a microarray approach and identified genes displaying differential expression upon histone demethylase inhibition across larval development. Furthermore, we used Gene Ontology to envision the putative physiological pathways related to these genes. To our knowledge, these results show the first evidence for histone methylation conservation and functional relevance in development outside vertebrates and ecdysozoans.

## 2. Materials and Methods 

### 2.1. Animals, Fecundation Assays, and Early Development in the Presence of the Jumonji Histone Demethylase Inhibitor, Methylstat

As previously described [25], in vitro fertilizations were realized with broodstock *Crassostrea gigas* specimens obtained from an oyster farm (Guernsey, GB) and the IFREMER experimental hatchery (Argenton, France). After gonad scarification, the gametes were filtered on a 100 µm mesh in order to remove the large debris. Spermatozoa (spz) and oocytes (oo) were collected on a 30 µm mesh. Oocytes were pre-incubated in filtered-sterile (0.22 µm) seawater (FSW) alone (Controls) or with 10 µM methylstat (Sigma, Taufkirchen, Germany) at 25 °C. Fertilizations were triggered by the addition of spermatozoa and were carried out in oxygenated FSW at 25 °C (500 oo/L; around 100 spz/oo). Embryos were left unattended until sampling, i.e., before fertilization for control oocytes, and 1 h post-fertilization (hpf) for 2–4 cells stage, 3 hpf for morulae, 6 hpf for blastulae, 9 hpf for gastrulae, 16 hpf for trochophore larvae, and 24 hpf for D larvae. Based on morphological and motility criteria before and after fixation using 70% ethanol, we assessed the developmental stages by microscopic observation. The development of methylstat–treated embryos was monitored in parallel using the same method, and animals were harvested after 6 (methylstat-6 h) and 24 (methylstat-24 h) hours, respectively. Embryos were concentrated by filtration on a 30 µm mesh then pelleted (500× *g*, 5 min). Dead embryos were not retained and went through the mesh. Supernatant was removed, then embryos were resuspended in TRI-reagent for RNA extraction (Sigma), directly stored at −80 °C (proteins extraction), or fixed (see Scanning Electron microscopy). 

### 2.2. Histones Extraction

As previously described [15], we have extracted oyster histones from the different embryonic stages by transferring them in TEB buffer (PBS containing 0.5% Triton X100, 2.10–3 mol/L PMS Fand 0.02% NaN_3_) at a concentration of 10^7^ cells/mL. Then, embryos have been lysed on ice for 10 min, and after centrifugation (900× *g*, 5 min, 4 °C), the cell lysate was transferred and pelleted (10,000× *g*, 1 min, 4 °C). We resuspended the cells in 3 volumes (225 μL/10^7^ cells) of extraction buffer (0.5 NHCl, 10% glycerol), and we incubated them on ice for 30 min before centrifugation (12,000× *g*, 5 min, 4 °C), resuspention in 8 volumes (600 μL/10^7^ cells) of acetone and incubation at −20 °C overnight. Finally, after centrifugation (15,000× *g*, 5 min), the pellet was dried and re-dissolved in distilled water (30–50 μL/10^7^ cells). We quantified the proteins using the Bradford method.

### 2.3. Histone Methylation Quantification

The H3K4, K9, K27, and K36 have been selected and mono-, di- and tri-methylation of each lysine residues were quantified in early stages of *Crassostre agigas* as in [15]. The purified oyster histone extracts (1–2 μg) (see above) were used for each methylation assay in fluorescent ELISA tests using specific antibodies (EpiQuik Global Pan-Histone Methyl (H3K4, 9, 27 and 36) Quantification Kit (Fluorimetric) (Epigentek, Farmingdale, NY, USA)). According the manufacturer’s instructions, we incubated the samples in a multi well plate coated with specific antibodies. After an incubation of 120 min at room temperature, samples were extensively washed and incubated for one hour at room temperature with a secondary antibody. After another extensive step of washing, the antibody binding was quantified by the addition of a fluorescent substrate and fluorescence measurement (Berthold Mithras 940 LE, excitation 530 nm and emission 590 nm). On the same plate, a standard curve, established with synthetic methyl-histone peptides, was used to measure histone methylation. The amount of mono-, di-, and tri-methylated H3K4-K9-K27 and K36 residues was calculated using the following formula:

Amount (ng/mg protein) = ((RFU (sample − blank))/ (Protein (μg) × slope)) × 1000

### 2.4. Scanning Electron Microscopy

Like described in [25], we fixed the samples during 1 h 30 min at 4 °C with 3.2% glutaraldehyde in cacodylate buffer 0.31 mol/L ph: 7.4 in presence of 0.25 mol/L sucrose. Afterwards, we have rinsed the cells three times in cacodylate buffer 0.4 mol/L pH: 7.4 in presence of 0.3 mol/L sucrose. After a sedimentation of several days on Thermanox^®^ (Waltham, MA, USA) coverslips coated with poly-l-lysine, the larvae were post-fixed with 1% osmium tetroxyde in cacodylate buffer 0.2 mol/L pH:7.4 in the presence of 0.36 mol/L sucrose (1 h, 4 °C protected from light). Then, embryos were washed in cacodylate buffer 0.4 mol/L pH: 7.4 in presence of 0.3 mol/L sucrose, before to be dehydrated in ethanol solutions with increasing concentrations (70–100%) and dried (CPD 030 LEICA Microsystem). The samples were coated with platinum and observed by scanning electron microscope (JEOL 6400F, JEOL, Peabody, MA, USA).

### 2.5. Microarrays

#### 2.5.1. RNA Amplification, Labeling, and Hybridization 

Sixteen pools of sample RNA (4 control-6 h, 4 methylstat-6 h, 4 control-24 h, 4 methylstat-24 h) were prepared for microarray analysis and processed as previously described [30]. According to the company’s instructions, we labeled 200 ng of total RNA using the Low Input Quick Amp labeling kit (Agilent, Santa Clara, California, USA). Then, we have purified the amplified RNA (aRNA) samples using the Qiagen’s RNA easy mini spin columns (Venlo, Netherlands). Afterwards, we measured the labeled aRNA concentration (between 200 and 500 ng/mL) and the dye incorporation (between 20 and 50 pmol/mg aRNA). The rates of dye incorporation and RNA amplification have been checked using an ND-1000 spectrophotometer (Nanodrop Technologies, Welmington, NC, USA). Using the Agilent’s Gene expression hybridization kit (5188–5242), we have performed the hybridization with 1.65 mg of aRNA samples labeled with Cy3. Subsequently, gene expression wash buffer solution (5188–5327; Agilent Technologies), stabilization and drying solutions (5186–5979; Agilent Technologies) have been used to wash the slides. Finally, we have scanned the slides using an Agilent Technologies G2565AA Microarray Scanner system at a 5 µm resolution.

#### 2.5.2. Correction and Normalization

Using the default/recommended normalization methods on the Agilent Feature Extraction software 6.1, we extracted and normalized the raw data. Then, we have generated a matrix of gene expression levels, where each column corresponds to one oyster treatment sample, and each row corresponds to a different gene. As in [31], we have logarithmically transformed and centered (relative to zero) the expression level of each gene. For interpretation [30], relative variations instead of absolute values were used.

#### 2.5.3. Data Analysis

To compare and identify statistically significant differentially expressed transcripts within control and methylstat larvae transcriptomes, we used Two factor ANOVA (time (6 and 24 h) and treatment (control and Methylstat 10^−5^ M)) with a p-value based on permutation (1000) of 0.05 and an α correction using TMeV 4.6.0 software (Institute for Genomic Research, Rockville, USA) [32,33]. Cluster analysis was employed to further determine the expression patterns occurring between treatments. Hierarchical clustering and K means clustering were performed using TMeV on the statistically significant transcripts [32,33]. Hierarchical clustering was used to group experimental samples together based on similarity of the overall experimental expression profiling [30]. Clusters were then compared using gene ontology analyses (see below). Histone methylation levels for each residue (H3K4, 9 and 27) were analyzed using One-way ANOVA (factor: development stage, *p* < 0.05 was considered significant). A student’s T test (*p* < 0.05 was considered significant) has been used to compare RT-qPCR data.

#### 2.5.4. Gene Ontology Analyses

Gene annotation analysis was carried out for all genes of the considered clusters, using the BLAST2GO program [34]. FASTA-formatted sequences representing all the transcripts within each considered cluster were uploaded to the program and BLASTX carried out against the Swiss-Prot database (http://blast.ncbi.nlm.nih.gov/Blast.cgi). Queries were annotated based on hit similarity and GO evidence codes [35]. Gene ontology (GO) analysis was improved and carried out with the GO annotation obtained from the GIGATON database [36]. The corresponding clustering genes were searched against this database using Blastn algorithm [37,38]. Enriched GO term was performed using the GO seq (v1.22.0) R package [39] with p-value calculated under hypergeometric method. GO term are considered significantly enriched with a *p*-value less than 0.05.

#### 2.5.5. RT-qPCR Validation of Microarray Analysis

Genes that displayed expression profiles representative of each cluster were randomly chosen to be measured by RT-qPCR in order to validate the accuracy of microarray experiments. RT-qPCR was performed as previously described [40]. Briefly, samples were extracted using Tri-Reagent (Sigma), and then RNA was purified using affinity chromatography (Nucleospin RNA II kit, Macherey-Nagel, Duren, Germany). Genomic DNA contamination was prevented by digesting the samples 30 min. with 1 U RQ1 DNAse (Promega, Madison, USA). Then, 250 ng of total RNA were reverse transcribed using 200 U of M-MLV RT (Promega) and 100 ng random hexamers. We diluted the resulting cDNAs, and the equivalent amount of 5 ng of starting RNA was assayed for the expression of the selected markers. The elongation-factor α (GenBank accession number: BAD15289) has been used as reference gene (average Ct (22.4) and STEDVA (0.5) between control/Methylstat and 6 h/24 h) [15,25,29]. We performed the SYBR-green quantitative PCR on an iCycler iQ apparatus (Bio-Rad) using 40 cycle (95 °C, 15 s; 60 °C, 15 s) reactions and GoTaq qPCR master mix (Promega). The primers efficiency was assessed for each pair, using standard curves on known DNA concentrations. All selected primer pairs displayed between 90% and 110% amplification efficiency. Their corresponding sequences are listed in (supplementary information Table S1). A melting curve and an end-point agarose gel electrophoresis followed by ethidium bromide staining were used to check the amplification of the target amplicon. A parallel amplification of the reference gene was carried out to normalize the marker transcript expressions. Using the following formula: N = 2^(CtRefgene − Ctmarker)^, the relative expression level of the selected markers was normalized to the reference gene. Absence of genomic DNA contamination has been checked, and water was used instead of cDNA as a negative control for amplification. All samples were analyzed in biological triplicates to establish the mRNA expression profile of the selected marker genes.

## 3. Results

### 3.1. Histone Methylation Exhibits Stage-Specific Patterns during Oyster Early Development

**Figure 1:**
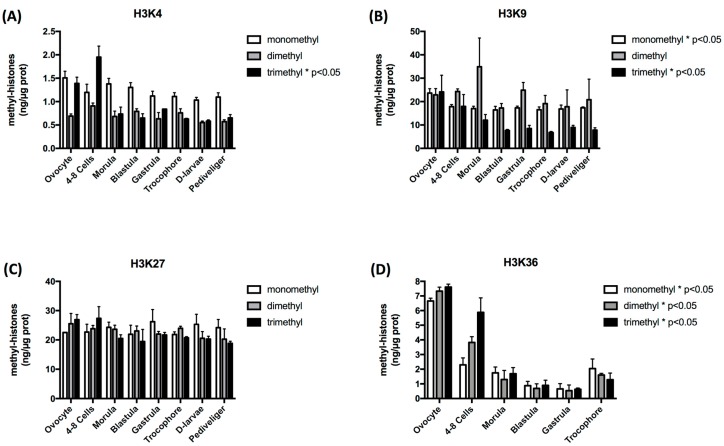
Histone methylation profiles of H3K4 (**A**), H3K9 (**B**), H3K27 (**C**) and H3K36 (**D**) during the early development of *Crassostrea gigas*. Asterisk in the legend indicates significant variation of the indicated mark along development A One-way ANOVA was used to determine whether methylation levels were statistically significantly different between development stage for each residue, and a *p*-value < 0.05 was considered significant.

The amount of methylation of histone H3 lysine 4, 9, 27, and 36 residues was observed to be in the same range overall, from ca. 0.5 to ca. 30 ng methyl histone/µg protein. However, some variations in the patterns of mono-, di- and tri-methylations could be observed across developmental stages, depending on the histone lysine residue and the number of methyl groups considered. Indeed, H3K4me1 and H3K4me2 remained stable whereas H3K4me3 decreased significantly after a maximum level observed in 4–8 cell embryos (*p* < 0.05). H3K9me1 and H3K9me3 also displayed a significant decrease, which was more marked up to the blastula stage and then remained stable. Finally, the global level of methylation on H3K36 also decreased especially until the blastula/gastrula stages before an increase in the trocophore stage. In contrast, no significant variation could be detected in the level of the methyl marks on H3K27 (Figure 1).

### 3.2. Methylstat-Induced Histone Hypermethylation is Correlated to Severe Developmental Alterations

**Figure 2:**
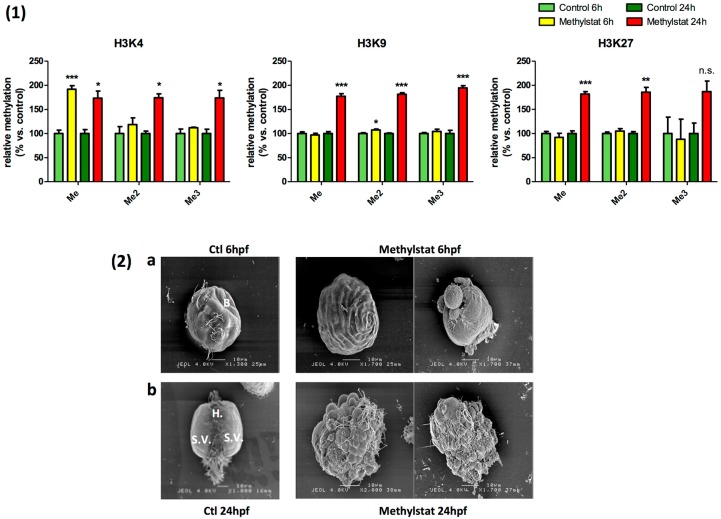
(**1**) Hypermethylation of histone lysine residues in presence of 10 µM Methylstat. Global H3K4 methylation increase in presence of methylstat, but only H3K4me1 and H3K4me3 are significant. Global H3K9 methylation increase in presence of methylstat, but only H3K9me2 is significant. H3K27me and H3K27me2 increase in presence of methylstat. Asterisk in the legend indicates significant variation of the indicated mark (One-way ANOVA; *p* < 0.05 was considered significant (<0.05 (*), <0.001 (**), <0.0001 (***)). (**2**) Abnormal development. Different phenotypes observed in control condition and under methylstat treatment at 6 h after fertilization (**a**) and 24 h after fertilization (**b**).

The treatment of embryos with methylstat induced an increase in histone methylation measured from 6 h after fertilization (hpf) (Blastula stage) and sustained till 24 hpf (Late Trochophore/D-larvae stage), consistent with the inhibitor effect of methylstat on JmjC-histone demethylases (Figure 2(1)). However, at 6 hpf, only H3K4me and H3K9me2 were significantly up regulated, with a 2-fold increase in H3K4me whereas H3K9me2 was only slightly increased. By contrast, at 24 hpf, the mono-, di- and tri-methylation of all the lysine residues examined was increased almost twice upon methylstat treatment, except H3K27me3 for which the apparent increase was not statistically significant (Figure 2(1)).

In correlation with the methylstat treatment, some developmental defects could be observed at 6 hpf already. While control embryos were at the Blastula stage, methylstat embryos exhibited a wide diversity of abnormal phenotypes (Figure 2(2)). They displayed an abnormal cell differentiation, and a reduced motility. Methylstat treated embryos also underwent some mortality (data not shown, <50%), while control embryos display a normal development. After 24 h of development in the presence of methylstat, the survival rate was significantly lower in treated embryos (data not shown). Larvae exposed to methylstat still presented abnormal phenotypes reflecting a failure to rescue a normal cell differentiation. Indeed, most of the cells looked alike with a round shape and ciliae, in contrast to the differentiated cells within normal trochophore and D-larvae animals (Figure 2(2)). 

### 3.3. Methylstat Treatment Induces Transcriptomic Variations

**Figure 3:**
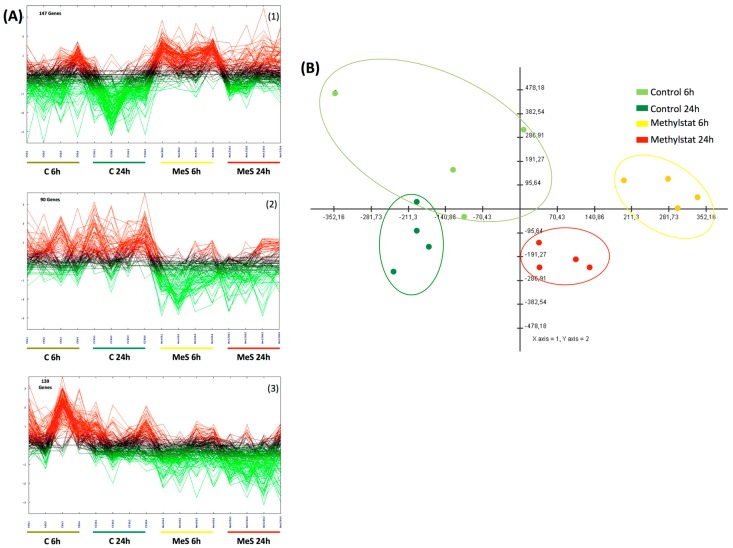
(**A**) Gene clustering. KMC clustering of the 376 genes differentially expressed upon methylstat exposure independently of development time. Three main clusters were discriminated (From the top to the bottom): (**1**) overexpressed in MeS-treated animals (147 genes), (**2**) underexpressed in MeS-treated animals (90 genes) and (**3**) underexpressed in MeS-treated 6hpf animals (139 genes). (**B**) Principal component analysis. The first three Principal component (PCs) explained 55.417% of the total variance of the 376 genes significantly differentially expressed between sample pools.

Sixteen pools of embryos, from control (4 control-6 h, 4 control-24 h) and Methylstat treated (4 methylstat-6 h, 4 methylstat-24 h) conditions, were used to perform a microarray experiment. After analysis of the microarray results with TMeV 4.6.0 software [32,33], a two factor ANOVA (time (6 and 24 h) and treatment (control and Methylstat 10^−5^ M)) with a p-value based on permutation (1000) of 0.05 and an α correction identified that 376 out of 31,918 analyzed ESTs were statistically differentially expressed between controls and treated animals. Hierarchical clustering using Pearson’s correlation grouped the 16 sample pools according to the treatment and the developmental time (data not shown). Biological interpretation of the data led us to group these clusters into 3 major gene expression profiles using K means clustering of genes according to similar expression patterns using Pearson’s correlation (Figure 3A). (I) Overexpressed genes in MeS-treated animals (147 genes, 39%, cluster 1) (Figure 3(A1)); (II) underexpressed genes in MeS-treated animals (90 genes, 25%, cluster 2) (Figure 3(A2)); (III) underexpressed genes in MeS-treated 6 hpf animals (139 genes, 37%, cluster 3) (Figure 3(A3)). The list of genes within each cluster is provided in supplementary data (Tables S2–S4). A Principal Component Analysis (PCA) was applied to all 376 genes of the sixteen sample pools (4 control 6 h, 4 control 24 h, 4 methylstat 6 h, 4 methylstat 24 h) to assess internal consistency of the whole transcriptional dataset and verify that the main variation in gene expression correspond specifically to treatment (Figure 3B). The first three Principal component (PCs) explained 58.5% of the total variance. The score plot obtained using the three first PCs is shown in Figure 3B. In this plot, similar transcriptional profiles cluster together, whereas significantly different samples appear more distant from each other. We observed a clear clustering of the different treatments and developmental times determined by microscopy and/or histone methylation pattern upon Methylstat treatment. 

### 3.4. Identification of the Putative Metabolic Pathways Implicating the Differentially Expressed Genes in Larvae Exposed to Methylstat

Homology searches (BLAST algorithm using default parameters and *E*-value < 10^−3^) of the differentially expressed genes led to the blast hits of 66% of them (246 out of 376) with 51% (193 out of 376) annotated and 15% (55 out of 376) identified as “unknown protein” or “hypothetical protein”. Thus, among the 147 overexpressed genes in MeS-treated animals (cluster 1), 47% (69 out of 147) are annotated (supplementary data Table S2), while 54% (49 out of 90 and 75 out of 139, respectively) have been identified in the two other clusters (underexpressed genes in MeS-treated animals (cluster 2), and genes underexpressed in MeS-treated 6 hpf animals (cluster 3), respectively) (supplementary data Tables S3 and S4). As an example, mRNAs overexpressed in MeS-treated animals include genes coding proteins implicated in cell cycle (Calm2 (CU991884), ANAPC4 (CU991170)), in cell proliferation and migration (TYRP1 (CU996139)), and gastrulation/embryonic development (CFC1 (CU988912)). Interestingly, among the underexpressed genes in MeS-treated animals, two genes were found to encode homeobox proteins (abd-B (AM857383) and Otp (CU991046)), which have been shown in mollusks to be critical regulators of early development [41]. Genes coding for proteins mediating the selective degradation of proteins such as UBA6 (FP005933) or UCHL3 (CU988028) also seemed to be regulated. However, only a limited number of sequences were assigned a GO, which makes precise interpretations of the physiological consequences of methylstat treatment rather speculative. Nevertheless, the GO enrichment using the GIGATON database [36] is consistent with the previously identified genes (supplementary data Tables S2–S4), and demonstrated that the differentially expressed transcripts within each cluster lie within a wide range of distinct GO categories among biological processes, molecular function and cell component that are not equally represented between clusters (Table 1; and supplementary data Table S5). Considering cluster 3 (genes underexpressed in MeS-treated 6hpf animals) for example, significantly enriched GO (*p* < 0.05) indicated a putative role in growth, map kinase activity and protein ubiquitination (Table 1; and supplementary data Table S5), while in cluster 1 (overexpressed genes in MeS-treated animals), GO categories are more implicated in ion transport or lipid biosynthesis (Table 1; and supplementary data Table S5).

### 3.5. Selected Marker Genes RT-qPCR Measurements Confirm Microarray Signals

**Figure 4:**
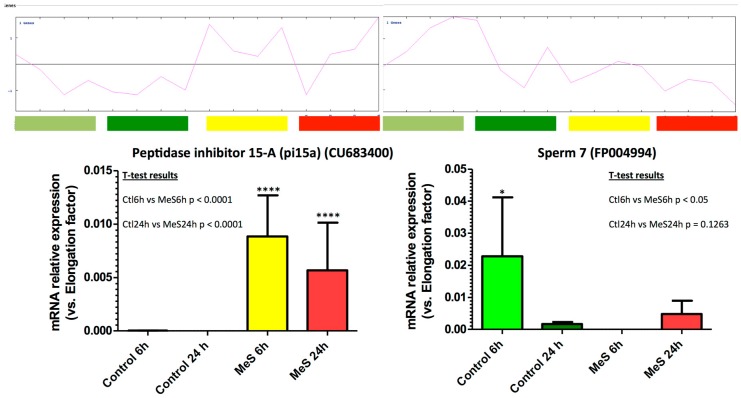
RT-qPCR measurements of mRNA expression of selected marker genes from the different clusters. Examples of cluster 1 (Peptidase inhibitor 15-a (pi15a) (CU683400), overexpressed in MeS treatment) and cluster 2 (Sperm7 (FP004994), underexpressed in MeS-treated 6hpf animals) are shown. For each transcript, a student’s T tests (*p* < 0.05 was considered significant) has been used to compare control 6 h (Ctl6h) vs. Methylstat sample 6h (MeS6h) and control 24 h (Ctl24h) vs. Methylstat sample 24 h (MeS24h). (****: *p* < 0.0001).

The marker genes selected for RT-qPCR expression measurements displayed mRNA level profiles that matched their microarray signal (Figure 4). These results further confirmed the validity of the microarray experiments, as previously described using the same arrays [30].

## 4. Discussion

Our study presents evidence of dynamic patterns of methylated histone residues and their potential importance for the development of the pacific oyster *Crassostrea* gigas by describing histone methylation levels of four lysine residues (H3K4, H3K9, H3K27, and H3K36) and the biological consequences of an exposure to an inhibitor of Jumonji histone demethylases during *C.gigas* early development. To our knowledge, this is the first report focusing on these mechanisms and their potential biological significance in lophotrochozoan development [14,15,16]. These results may contribute to a better understanding of the evolution of epigenetic regulation that can be applied to research fields such as aquaculture or response of marine organisms to global changes. 

In this work, we show that some histone methyl marks (H3K4me3, HEK9me1, H3K9me3, and H3K36me) seem to be highly regulated during oyster early development, while others (H3K4me, H3K4me2, H3K9me2, and H3K27me) appear to be more stable. Thus, the high level of H3K4me3 in oocytes and 4-8 cells followed by its reduction and the global decrease of H3K9me1/3 and H3K36me/me2/me3 could be reflective of the pre-patterning of the activation of early zygotic development genes in the oyster [27]. This reminds of the global erasure of histone marks observed during the reprogramming event in the zebrafish and the mouse [42,43,44,45]. However, as for DNA methylation [25,46], patterns of histone marks reveal both similarities and differences with vertebrates and highlight the distinct reprogramming events happening among species [43,47,48,49]. Indeed, global methylation of H3K27 appears to be stable during oyster early development, while in vertebrates, H3K27me3 is erased after fertilization before being deposited again in later stages [43,44,45]. 

The patterns of histone marks that are generally associated with transcriptional activation (H3K4me3 and H3K36me3) might reflect high chromatin accessibility at the very beginning of oyster development. In characterized model organisms, the chromatin is indeed more accessible during the very early stages of embryogenesis before it gradually changes to a more compact conformation [42,47]. Interestingly, H3K4me is more stable and remains high throughout the larval life. This suggests a potential role of H3K4me in the maintenance of embryonic stem cells [50] and is partially reminiscent of the H3K4me dynamics in the mouse [51]. Furthermore, as in vertebrates [3,45], the stable pattern of H3K27me3 associated with dynamic levels of H3K4me1/3 might indicate a conserved role for these histone residues in maintaining, respectively, “poised” enhancers and promoters along oyster development. Therefore, the methylation of H3K4 in the oyster might have distinct roles depending on the number of methyl groups and their position [45] illustrating a very precise tuning of the transcriptional status of the chromatin during the development of this representative of lophotrochozoan organisms. The demethylation of H3K9 is indicated by a dramatic decrease in the H3K9me3 mark. However, due to the absence of an expected subsequent increase in H3K9me1 and me2 levels, it is speculated that trim ethylated H3K9 residues become completely demethylated locally, rather than undergoing a genome-wide demethylation during development. These two mechanisms do not necessarily exclude one another and could eventually be observed on other histone residues such as H3K4 in our study. This might also reflect differential histone lysine methylation regulation depending on the chromatin loci examined. In summary, the observed methylation profiles could suggest gene-specific regulation of transcription, but more in-depth studies are required to decipher this point. Indeed, it is unknown whether the relationship between histone methyl marks, chromatin structure and transcriptional regulation is conserved between oysters and vertebrates. Nevertheless, it is likely that these marks may have direct, indirect, *cis*, and *trans* effects on transcription, possibly through both chromatin conformation and regulation of transcription activators or repressors. Such molecular mechanisms clearly require further investigations and may be hard to detangle but probably explain why we observe the upregulation of some genes together with the downregulation of others. Interestingly, the potential biological relevance of H3K9 methylation level is consistent with the observed peaks in both H3K9me2 and DNA methylation at the morula stage [25], which could indicate a conservation of the epigenetic regulation of pluripotency genes in stem cells [6,7]. H3K27 methylation is generally considered a repressive mark but was not significantly modified during oyster development. Together, our results might suggest specific chromatin landscapes outcomes of the different forms of methyl histones in the oyster, as well as the conservation of the H3K27 potential bivalent function when associated to H3K4me1/me3 [3,45]. Finally, high levels of H3K36me3 in oyster oocytes and 4-8 cell embryos may play a role in the de novo DNA methylation ongoing until the morula stage [25]. In vertebrates, this mark might help recruiting DNMT3a/b, the enzymes that are responsible for the de novo DNA methylation during the reprogramming event [52]. Generally associated to transcriptional activation [42,47], the global decrease of H3K36 methyl marks during *C. gigas* development might indicate a decreased chromatin accessibility in the late embryogenesis as characterized in model organisms [42,47]. 

The biological significance of histone lysine methylation during oyster development was further assessed by the effect of a selective inhibitor of Jumonji C domain-containing histone demethylases (JmjC), Methylstat. Methylstat was shown in mammalian cells to specifically inhibit JmjC enzymes at the micromolar range at least 100 times over other histone demethylases [53]. Parallel to the great increase in the methylation of H3K4, H3K9, and H3K27 in treated cells [53] or in drosophila/mouse JmjC loss of function mutants [47,54], oysters exposed to Methylstat display dramatic histone hypermethylation together with developmental defects. Interestingly, while no significant variations are identified for H3K27 in normal oyster development, a significant hypermethylation is observed at 24 h on H3K27me1 and H3K27me2 in presence of Methylstat. This result might reflect developmental consequences of Methylstat treatment beyond, or even independent of, the alteration of the normal developmental histone methylation pattern and dynamics. Therefore, it is possible that the Methylstat-induced H3K27 hypermethylation may have effects independently of the developmental stage itself but rather related to the overall cell physiology. However, ELISA tests give only global variations without picturing the precise localization of each methylation mark in the genome. Therefore, we cannot exclude that changes in H3K27 (and others) methylation levels and position at specific loci or between differentiated cell types and embryonic stages may be masked by similar levels assayed at the whole organism scale. Besides, the hypermethylation of other residues together with the alteration gene expression at 6 hpf already in the presence of Methylstat might affect the correct expression and/or biochemical activity of H3K27 “writers, readers and erasers” as observed in some human cancers [55]. These results strongly suggest the implication of histone methylation patterns in the developmental control. 

We have previously characterized a conserved Jmj gene family in the oyster [29], and we also have identified the actual presence of JmjC-domain containing proteins in oyster embryos using LC/MS-MS (data not shown). This strongly suggests the conservation of the demethylase activity of oyster JmjC proteins [29] and that the observed hypermethylation is mediated by the specific inhibition of such activity by Methylstat. Nevertheless, it cannot be strictly excluded that the observed phenotypes may partially result from methylstat toxicity. However, while cell-culture models are not easily comparable with oyster embryos, no acute toxicity could be observed in Methylstat-treated cells even at higher doses [53]. Furthermore, the treatment led to a dramatic increase (ca. twice for all marks and all residues examined at 24 hpf) in histone methylation. This pattern further suggests that the development defects might arise from altered gene transcription outcomes, which likely originate in histone methylation defects. Because the different phenotypes associated to histone hypermethylation consist in apparent abnormal cell differentiation and larval development as soon as 6 h after fecundation, we hypothesized that they were supported by transcriptomic alterations. 

Microarray studies revealed that the deleterious methylstat treatment-induced histone hypermethylation that influenced larval transcriptomes. This study led to the determination of the genes differentially expressed in association with the abnormal histone methylation pattern induced by Methylstat. *In silico* analyses indicated that the aforementioned genes belong to a wide range of distinct ontology categories of biological processes, molecular functions, and cell components. Therefore, it has not been possible to precisely assign accurate physiological pathways to the different gene expression clusters, leading to the assumption that the methylstat treatment transcriptomic consequences are little specific if not at all. Therefore, histone hypermethylation probably affected the whole genome in our experiments. Despite these results strongly suggesting the critical influence of histone methylation patterns during oyster development, the precise underlying mechanisms remain unknown because many sequences could not be assigned a Gene Ontology annotation (58% of the sequences). As a consequence, further interpretations are too speculative, despite some of the differentially expressed genes were shown or strongly suspected to be of relevance in a developmental context. Examples include *Homeobox* genes participating in regionalization, patterning, and cell differentiation during embryogenesis and organ development [5], which are conserved in mollusks [41]. In addition, genes implicated in RNA splicing and gastrulation, such as *syf2* (CU988555), in cell differentiation, such as *LTBP4* (AM861255), the *ubiquitin carboxyl-terminal hydrolase isozyme l3* (CU988028) and the *neurogenic locus notch homolog protein 1* (*NOTCH1*) (AM855770), and also genes involved in cell structure, such as *CFC1* (CU988912), or cell cycle progression, such as *ube2ib* (CU990470), are regulated. These elements, despite being scarce, indicate profound physiological alterations of the cell metabolism associated to histone lysine hypermethylation in developing oyster embryos and remind of the developmental arrest and deregulated transcriptional patterns observed in ecdysozoan and mammals associated with hypermethylation of H3K4, K9, and K27 residues [47,54,56]. Furthermore, even if the relationship between zygotic transition and chromatin remodeling remains unclear in mammals [47], evidence of a crucial role in this process for H3K4me1, HEK4me3, H3K9me2, H3K9me3, and H3K27 starts to accumulate for different organisms [42,47,52]. Thus, in zebrafish [47], H3K4me1 prevents DNA methylation thereby allowing the transcription following zygotic transition, while in mammals H3K9me2 protects DNA from demethylation [52]. In this study, a hypermethylation of H3K4me1 is observed at 6 hpf, and a global hypermethylation of H3K9 residues appears at 24 h. As demonstrated in [25], DNA methylation crucially accumulated until the morula/blastula stages and drops in gastrula. Moreover, it is shown that DNA methylation regulates precisely the transcription of many genes [27] such as the histone demethylase *JmjD6* [25] or different *Hox* genes [28] in *C*. *gigas*. Interestingly, some *HOX* genes have been found downregulated in presence of Methylstat, while a hypermethylation of H3K27me1/me2 is observed. This reminds partially of the situation in mouse where embryonic stem cells present little enrichment in H3K27me3 particularly in *HOX* genes and where H3K27 methylation is necessary to prevent aberrant H3K27 acetylation during the zygotic transition [47]. Finally, many ubiquitin hydrolases and transcriptional factors have been found deregulated in oyster embryos exposed to Methylstat. Because the ubiquitin proteasome system is shown to regulate maternal protein degradation, during the transcriptional activation required for the MZT [42], such pathway might also be important in the oyster.

Like DNA methylation [25,27], histone modifications are suspected to impact early genes expression [5], in line with what is known in vertebrates [47]. Nevertheless, only very little is known about histone modifications [14] and almost nothing on methylation in lophotrochozoan [15]. Further studies are needed to elucidate the importance of H3K36 but also for the other histone marks. While we have identified the JmjC domain at the protein level in oyster embryos, the precise determination of the actual level of each oyster Jmj protein and of its enzymatic activity should be further investigated. For instance, in vitro demethylation activity assays of recombinant oyster Jmjs would be helpful but lie beyond the scope of the present study. Furthermore, mechanisms of transcriptional regulation by histone methylation remain poorly understood in oyster [15,25,29]. Thus, genome-wide analysis of these epigenetic marks and chromatin accessibility using ATAC-seq, together with the elucidation of cell-specific spatio-temporal expression patterns of the histone methylation and the candidate genes, should be undertaken using high-throughput techniques such as ChIP-seq with specific methyl-histones antibodies. These results could provide extremely interesting insights into the epigenetic regulation of development in the oyster and more generally in lophotrochozoans. 

## 5. Conclusions

This study demonstrates the presence and the potential importance of histone methylation in *Crassostrea gigas*. The evolutionary conservation of this epigenetic mark and its stage-specific patterns during development suggest an important biological function consistent with the role of these mechanisms in other species. Our results bring the first evidence for regulation of histone methylation in lophotrochozoans, further questioning a possible conservation of theses epigenetic marks in this group.

## Figures and Tables

**Table 1:** Gene ontology (GO) terms of the 376 genes differentially expressed.

	GO-ID	GO-Term	GO-Class	*p*-Value
Cluster 1	0005890	Sodium:potassium-exchanging ATPase complex	CC	0.0027
	0044183	Protein binding involved in protein folding	MF	0.0027
	0006814	Sodium ion transport	BP	0.0031
	0004126	Cytidine deaminase activity	MF	0.0054
	0009972	Cytidine deamination	BP	0.0054
	0080019	Fatty-acyl-CoA reductase (alcohol-forming) activity	MF	0.0054
	0030126	COPI vesicle coat	CC	0.0080
Cluster 2	0005882	Intermediate filament	CC	0.000045
	0004952	Dopamine neurotransmitter receptor activity	MF	0.0015
	0005198	Structural molecule activity	MF	0.0022
	0050829	Defense response to Gram-negative bacterium	BP	0.0030
	0050830	Defense response to Gram-positive bacterium	BP	0.0030
	0000087	Mitotic M phase	BP	0.0045
	0008250	Oligosaccharyltransferase complex	CC	0.0045
	0004579	Dolichyl-diphosphooligosaccharide-protein glycontransferase	MF	0.0060
	0005887	Integral component of plasma membrane	CC	0.0089
	0016641	Oxidoreductase activity, acting on the CH-NH2 group of donors, oxygen as acceptor	MF	0.0089
Cluster 3	0006511	Ubiquitin-dependent protein catabolic process	BP	0.0020
	0003868	4-hydroxyphenylpyruvate dioxygenase activity	MF	0.0031
	0003922	GMP synthase (glutamine-hydrolizing) activity	MF	0.0031
	0006177	GMP biosynthetic process	BP	0.0031
	0004879	RNA polymerase II transcription factor activity, ligand-activated sequence-specific DNA binding	MF	0.0062
	0010309	Acireductone dioxygenase (iron(II)-requiring) activity	MF	0.0062
	0016701	Oxydoreductase activity, acting on single donors with incorporation of molecular oxygen	MF	0.0062
	0030833	Regulation of actin filament polymerization	BP	0.0062
	0000220	Vacuolar proton-transporting V-type ATPase, V0 domain	CC	0.0092
	0004427	Inorganic diphosphatase activity	MF	0.0092
	0006206	Pyrimidine nucleobase metabolic process	BP	0.0092
	0006796	Phosphate-containing compound metabolic process	BP	0.0092
	0016154	Pyrimidine-nucleoside phosorylase activity	MF	0.0092
	0016462	Pyrophosphatase activity	MF	0.0092
	0019509	L-methionine biosynthetic process from methylthioadenosine	BP	0.0092

Cluster 1: The seven most enriched terms and the corresponding p-value (hypergeometric method) for enrichment are given among the overexpressed genes in MeS-treated animals (147 genes). Cluster 2: The ten most enriched terms and the corresponding *p*-value (hypergeometric method) for enrichment are given among the underexpressed genes in MeS-treated animals (90 genes). Cluster 3: The fifteen most enriched terms and the corresponding *p*-value (hypergeometric method) for enrichment are given among the underexpressed genes in MeS-treated 6hpf animals (139 genes). CC (Cellular Components), BP (Biological Process), MF (Molecular Function).

## Supplementary Materials

The following are available online at https://www.mdpi.com/2073-4425/10/9/695/s1, Table S1: qPCR Primers, Table S2: Cluster 1_147 genes, Table S3: Cluster 2_90 genes, Table S4: Cluster 1_139 genes, Table S5 Go term enrichment.
