# Supplementary material for: Histone Methylation Participates in Gene Expression Control during the Early Development of the Pacific Oyster Crassostrea gigas
Source: Genes (Basel). 2019 Sep 10;10(9):695. doi: 10.3390/genes10090695 (PMC6771004; doi:10.3390/genes10090695)
Supplement: Supplementary file 1 [file genes-10-00695-s001.zip › genes-576805-supplementary/Supplementary_data/Supplementary_Data_1_Table_1_qPCR_Primers.rtf]

Spermatogenesis-associated protein 7_L  5’-AAACCAAAGCCGAGAGTCAA-3’ Spermatogenesis-associated protein 7_R  5’-ATGTCCAGTCTGGGGACAAC-3’ Peptidase_Inhibitor_15_A_L  5’-GTTTTCATCTCCGACGTGCT-3’ Peptidase_Inhibitor_15_A_R  5’-AAATGCAGAAAGGCTTGTGG-3’ Elongation_Factor_L 5’-ACCACCCTGGTGAGATCAAG-3’ Elongation_Factor_R 5’-ACGACGATCGCATTTCTCTT-3’ 
