# Supplementary material for: Histone Methylation Participates in Gene Expression Control during the Early Development of the Pacific Oyster Crassostrea gigas
Source: Genes (Basel). 2019 Sep 10;10(9):695. doi: 10.3390/genes10090695 (PMC6771004; doi:10.3390/genes10090695)
Supplement: Supplementary file 1 [file genes-10-00695-s001.zip › genes-576805-supplementary/Supplementary_data/Supplementary_Data_1_Table_2_Cluster_1_147_genes.pdf]

Accession Number	Gene Name	Putative biological function (source UniProt)
CU988981	Alpha-N-acetylgalactosaminidase (**NAGA**)	Glycolipid and carbohydrate catabolic process
FP009095	Protein **MMS22**	Genome integrity during DNA replication
CU991170	Anaphase-promoting complex subunit 4 (**ANAPC4**)	Positive regulation of mitotic metaphase/anaphase transitions
AM864639	Vacuolar-sorting protein **SNF8**	Endosomal transport and transciptionnal regulation
CU991506	Tripartite motif-containing protein 2 (**TRIM2**)	Protein polyubiqutination
AM857976	Contactin-5 (**CNTN5**)	Cell adhesion during nervous system development
FP001149	Phenylserine dehydratase	Gluconeogenesis
AM854656	Reversion-inducing cysteine-rich protein with Kazal motifs (**RECK**)	Embryonic morphogenesis, cell migration, regulation of the Wnt pathway
CU995707	Vacuolar ATPase assembly integral membrane protein **VMA21**	Regulation of ATPase activity
AM861623	Solute carrier family 13 member 2 (**SLC13A2**)	Transport of sodium ions and dicarboxylate
CB617395	Integrin beta-like protein A (**ITGBL1**)	Cell adhesion
CU992985	Delta(14)-sterol reductase-like (**DBR2**)	Metabolic process
FP011715	26S proteasome non-ATPase regulatory subunit 5 (**PSMD3**)	Maintenance of protein hometostasis, cell cycle progression
AM861441	Asteroid-like protein 1 (**ASTE1**)	DNA repair
FP002068	Mitochondrial fission factor (**MFF**)	Mitochondrial fission
CU988912	Cryptic protein (**CFC1**)	Gastrulation, Heart development, anatomical structure development
CU988028	Ubiquitin carboxyl-terminal hydrolase isozyme L3 (**UCHL3**)	Ubiquitin-dependent protein catabolic process
AM862952	Fanconi anemia core complex-associated protein 20 (**FAAP20**)	DNA damage, DNA repair
CU986965	Collagen alpha-1(I) chain (**COL1A1**)	Embryonic development and ossification
CU991884	Calmodulin-2 (**Calm2**)	Cell cycle progression
AM865788	Cytidine deaminase (**CDA**)	Cell growth
AM868621	Protein **ARV1**	Sterol and lipid metabolism, transport
CU986603	Heat shock 70 kDa protein 12A (**HSPA12A**)	ATP binding
CU996139	Tyrosinase-like protein 1 (**TYRP1**)	Cell cycle proliferation

FP011792	Phosphatidylinositol N-acetylglucosaminyltransferase subunit P (**PIGP**)	GPI-anchor biosynthesis
ES789933	Coatomer subunit gamma-2 (**COPG2**)	ER-Golgi transport, Protein transport, Transport
FP008417	Neurochondrin Iso 2 (**NCDN**)	Signaling transduction
AM867236	Filamin-A-interacting protein 1 (**FILIP1**)	Cell migration
CU995344	Tetraspanin-3 (**TSPAN3**)	Cell proliferation and migration
AM862482	Protocadherin alpha-C2 (**PCDHAC2**)	Cell adhesion
CU683837	Senescence associated protein (**DIN1**)	Sencescence
CU997478	Carnosine synthase 1 (**CARNS1**)	Carnosine biosynthetic process
CU990242	Peptidyl-prolyl cis-trans isomerase **FKBP4**	Protein folding
CU999062	Tripartite motif-containing protein 2 (**TRIM2**)	Protein polyubiqutination
AM866619	Peroxidasin homolog (**PXDN**)	Hydrogen peroxyde
AM855549	Sodium-independent sulfate anion transporter (**SLC26A11**)	Anion exchange, Ion transport, Transport
AM865586	Tripartite motif-containing protein 75 (**TRIM75**)	Metal-binding
CU990665	Sodium/potassium-transporting ATPase subunit beta (**ATP1B1**)	Cell adhesion, Sodium/potassium transport
AM864143	Nuclear pore complex protein **Nup107**	mRNA transport, Protein transport, Translocation, Transport
FP007022	Small integral membrane protein 7 (**Smim7**)	Unknown
FP005820	UPF0582 protein C13orf37, mitotic spindle organizing protein 1 (**MZT1**)	Gamma-tubulin Complex localization
FP006374	Eukaryotic translation initiation factor 2-alpha kinase 3 (**EIF2AK3**)	Stress response, Translation regulation, Unfolded protein response
FP008354	Tyrosinase	Cell population proliferation
FP010483	Focadhesin Iso 2 (**Focad**)	Potential tumor suppressor
EX956409	Tripartite motif-containing protein 3 (**TRIM3**)	Protein transport, Transport, Nervous system development
CU999647	Interferon-induced protein 44 (**IFI44**)	Immune response
FP009925	GRIP1-associated protein 1 (**GRIPAP1**)	Protein transport, Transport
CX068921	Perilipin-2 (**PLIN2**)	Development and maintenance of adipose tissue
CU993737	Sodium- and chloride-dependent glycine transporter 2 (**Slc6a5**)	Neurotransmitter transport, Symport, Transport
CU991458	Dystrophin (**DMD**)	Muscle organ development
AM865975	LON peptidase N-terminal domain	Protein polyubiqutination

	and RING finger protein 3 Iso 1 (**LONRF1**)	
CU997996	Nucleoside-triphosphate phosphatase	Cellular response
AM864917	Tyrosine-protein kinase **BTK**	Adaptative immunity and transcription regulation
CU683400	Peptidase inhibitor 15-A (**pi15a**)	Multicellur organsim development
FP002317	Zinc finger protein 511 (**ZNF511**)	Transcription regulation
FP003562	Tripartite motif-containing protein 3 (**TRIM3**)	Protein transport, Transport, Nervous system development
CU991980	Mitochondrial 2-oxodicarboxylate carrier (**SLC25A21**)	Transport
FP009692	Transmembrane protein 19 (**TMEM19**)	Transport
AM856286	DNA-directed RNA polymerases I, II, and III subunit (**RPABC3**)	Transcription
FP012155	39S ribosomal protein L1 (Mitochondrial) (**MRPL1**)	RNA-binding
CU990714	1-phosphatidylinositol 4,5-bisphosphate phosphodiesterase eta-2 (**PLCH2**)	Neural development
CX739579	Short-chain collagen C4	Unknown
CU997185	shisa-5	Apoptosis
CU990470	SUMO-conjugating enzyme UBC9-B (**ube2ib**)	Cell cycle, Cell division, protein sumoylation
AM861301	Kinesin KIF20B	
FP000402	Regulator of rDNA transcription protein 15 (**RRT15**)	Transcription regulation
FP005094	Threonylcarbamoyladenosine tRNA methylthiotransferase (**CDKAL1**)	tRNA processing
AM863112	Gigasin-6	Protease function
AB288344	Lysozyme 2 (**Lyz2**)	Lysozyme activity
