# Supplementary material for: Histone Methylation Participates in Gene Expression Control during the Early Development of the Pacific Oyster Crassostrea gigas
Source: Genes (Basel). 2019 Sep 10;10(9):695. doi: 10.3390/genes10090695 (PMC6771004; doi:10.3390/genes10090695)
Supplement: Supplementary file 1 [file genes-10-00695-s001.zip › genes-576805-supplementary/Supplementary_data/Supplementary_Data_2_Table_3_Cluster_2_90_genes.pdf]

Accession Number	Gene Name	Putative biological function (source UniProt)
AM856044	**Tctex1** domain-containing protein 2	Cilium assembly
CX068923	valine-rich protein Iso 4	Metabolism
CU684113	Retrograde protein of 51 kDa (**RGP51**)	Unknown
CU993915	Tubulin polyglutamylase complex subunit 1 (**TPGS1**)	Differentiation, Spermatogenesis
AJ565452	Beta-1,3-galactosyltransferase 1 (**B3GALT1**)	Protein glycosylation
CU996496	Glycine-rich cell wall structural protein (**GRP-1**)	Cell wall biogenesis/degradation
AM865428	Nucleoside-triphosphate phosphatase	DNA and RNA synthesis
FP010217	Sodium/potassium/calcium exchanger 2 (**SLC24A2**)	Ion transport
CU989510	Pyruvate carboxylase	Metabolism
FP002044	Sentrin-specific protease 1 (**SENP1**)	Ubl conjugation pathway
CU996825	Phosphatidate phosphatase (**app1**) Iso2	Lipid metabolism
FP002409	Glutaredoxin-like protein C5orf63 homolog	Electron transport
CX069021	IgGFc-binding protein (**FcγBP**) Iso2	Maintenance
FP007188	N-alpha-acetyltransferase 25, NatB auxiliary subunit (**NAA25**)	N-terminal peptidyl-methionine acetylation, Cell cycl progression
CU993728	Protein **CLN8**	Cell proliferation, metabolism
FP001871	Nuclear hormone receptor **E75**	DNA Binding
AM856297	Tripartite motif-containing protein 2 (**TRIM2**)	Ubl conjugation pathway
FP011648	Monocarboxylate transporter 2 (**SLC16A7**)	Symport, transport
FP002939	23S rRNA (guanine(2445)-N(2))-methyltransferase	Unknown
AM854490	EGF-like repeat and discoidin I-like domain-containing protein 3 (**EDIL3**)	Cell adhesion
CU996619	Zinc finger protein 593 homolog (**Y56A3A.18**)	ribosomal large subunit export from nucleus
AJ565497	Tubulin polymerization-promoting protein family member 3 (**TPPP3**)	Bind tubulin, cell proliferation, mitosis
CU681484	Hemicentin-2 (**Hmcn2**)	Response to stimulus
FP001130	Retinol dehydrogenase 11 (**RDH11**)	Retinoid metabolic process
FP009647	Solute carrier family 23 member 2 (**SLC23A2**)	Symport, transport
AM854697	Ectoine hydroxylase	2-oxoglutarate-dependent dioxygenase activity

CU999928	NAD(P) transhydrogenase subunit alpha (**pntA**)	NADPH regeneration, proton transmembrane transport
CU986550	Cytochrome P450 26A1 (**CYP26A1**)	Organ development, metabolism
CU994988	Protein **PIF**	Chitin metabolic process
FP009041	Cation-independent mannose-6-phosphate receptor (**IGF2R**)	Organ development, transport
CU994773	Neurensin-1 (**Nrsn1**)	Nervous system development
CU999098	Ribonuclease PH (**Rph**)	rRNA et tRNA processing
FP091108	Dopamine receptor 1 (**DOP1R1**)	Response to stimulus
AM861792	Dolichyl-diphosphooligosaccharide (**Rpn1**)	Cellular protein modification process
FP008253	Big defensin 3 (**BigDef3**)	Antimicrobial peptide
AM855042	Muscle M-line assembly protein **unc-89**	Cellular protein localization
CU684359	Valine-rich protein Iso 4	Metabolism
AM865375	FH2 domain-containing protein 1 (**FHDC1**)	Cillium assembly, Golgi Ribbon formation
FP009859	Pilus assembly protein **PilP**	Unknown
ES789253	Translation initiation factor IF-2 Iso1 (**InfB**)	Protein biosynthesis
FP004396	Mantle protein	Unknown
FP007080	Hydroxylamine reductase (**hcp**)	Hydroxylamine catalyzation
AM857383	Homeobox protein abdominal-B (**abd-B**)	Embryonic development, germ cell migration
CU684641	D-aspartate oxidase (**DDO**)	Aspartate metabolic/catabolic processes
FP005933	Ubiquitin-like modifier-activating enzyme 6 (**UBA6**)	Ubl conjugation pathway
AM858253	Histone RNA hairpin-binding protein (**Slbp**)	mRNA processing, both maternal and zygotic proteins play an essential and vital function for development
FP007567	Exosome complex component RRP46 (**EXOSC5**)	tRNA processing
CX069312	Retrograde protein of 51 kDa Iso2 (**RGP51)**	Unknown
AM863498	Tripartite motif-containing protein 3 (**TRIM3**)	Ubl conjugation pathway
