# Supplementary material for: Histone Methylation Participates in Gene Expression Control during the Early Development of the Pacific Oyster Crassostrea gigas
Source: Genes (Basel). 2019 Sep 10;10(9):695. doi: 10.3390/genes10090695 (PMC6771004; doi:10.3390/genes10090695)
Supplement: Supplementary file 1 [file genes-10-00695-s001.zip › genes-576805-supplementary/Supplementary_data/Supplementary_Data_3_Table_4_Cluster_3_139_genes.pdf]

Accession Number	Gene Name	Putative biological function (source UniProt)
FP008154	Peptide-N(4)-(N-acetyl-beta-D-glucosaminyl)asparagine amidase F GNFase F (**Ngl**)	Unknown
FP010154	Complement C1q-like protein 4 (**C1QL4**)	Negative regulation of ERK1 and ERK2 cascades, adipocyte differentiation
AM857325	Neuronal acetylcholine receptor subunit alpha-6 (**CHRNA6**)	Ion transport
CU991479	Kyphoscoliosis peptidase (**KY**)	Muscle growth
CU988470	Fos-related antigen 1 (**FOSL1**)	Embryonic development, cell proliferation and cycle, response to a stimulus
CU984081	Tripartite motif-containing protein 2 (**Trim2**)	Ubl conjugation pathway
CU997410	Vacuolar protein sorting-associated protein 28 homolog (**VPS28**)	Protein transport, Transport
CU989588	Dual specificity protein phosphatase 14 (**DUSP14**)	Involved in the inactivation of MAP kinases
CU990751	S-phase kinase-associated protein 1 (**SKP1**)	histone H2A monoubiquitination, Cell cycle, Ubl conjugation pathway, Wnt signaling pathway
AM866181	Cytochrome P450 3A4 (**CYP3A4**)	Lipid metabolism, Steroid metabolism, Sterol metabolism
CU990917	Protein **psiR**	Unknown
CU986392	Actin-related protein 2/3 complex subunit 2 (**ARPC4**)	Response to DNA damage by promoting nuclear actin polymerization
AM863962	Nacrein-like protein P2	Regulator for calcification in the shells of mollusks
AM868879	Protein disulfide-isomerase **TMX3**	Cell redox homeostasis
FP000321	40S ribosomal protein S6 (**Rps6**)	Cell growth and proliferation
CU987157	Transmembrane protein 233 (**TMEM233**)	Unknown
AM853237	**CD109** antigen Iso2	Cell proliferation, negative regulation of protein phosphorylation
CU988311	COP9 signalosome complex subunit 6 (**COPS6**)	Post-translational protein modification, nucleotide-excision repair, DNA damage recognition
CU991460	Electroneutral sodium bicarbonate	Ion transport, cell pH

	exchanger 1 (**SLC4A8**)	regulation and survival
CU996633	Alpha-crystallin B chain Iso2 (**CRYAB**)	Negative regulation of transcription and apoptotic process
CU986548	Putative tyrosinase-like protein **tyr-3**	Melanin biosynthetic process
FP010231	Matrix metalloproteinase (**MMP**)	Collagen degradation, regulation of cell migration and DNA dammage
AM866267	Toll-like receptor 3 (**TLR3**)	Immunity, Inflammatory response, Innate immunity
AM861255	Latent-transforming growth factor beta-binding protein 4 (**LTBP4**)	Development, cell differentiation, cell growth
AM855778	L-xylulose reductase (**DCXR**)	Carbohydrate metabolism, Glucose metabolism, Xylose metabolism
CU992874	Polyamine-modulated factor 1-binding protein 1 (**PMFBP1**)	Spermatogenesis
AM859820	Interferon-induced protein 44 Iso2 (**IFI44**)	Immune response
CU987577	Peptidyl prolyl cis-trans isomerase B (**PPIB**)	Developement
CU684943	Period circadian protein Iso2 (**Per**)	Biological rhythms
CU995019	GMP synthase (**GMPS**)	Cell division, GMP biosynthesis, Purine biosynthesis
CU984299	Ectoine hydroxylase	unknown
CU987464	Tumor protein p53-inducible protein 11 Iso3 (**Tp53I11**)	Negative regulation of cell population proliferation
AM865696	Beta-1,4-galactosyltransferase **galt-1**	N-glycan processing, protein galactosylation
CU683571	Tripartite motif-containing protein 2 (**TRIM2**)	Ubl conjugation pathway
FP010896	Cytochrome P450 2J6 (**CYP2J6**)	Metabolism
CU984444	Ankyrin repeat and IBR domain-containing protein 1 (**ANKIB1**)	Ubl conjugation pathway
CU999022	N-acetylglucosamine-1-phosphodiester alpha-N-acetylglucosaminidase (**NAGPA**)	Unknown
FP002052	Thymidine phosphorylase (**TYMP**)	Angiogenesis, Chemotaxis, Cell Differentiation
CU984459	Electrogenic sodium bicarbonate cotransporter 1 (**SLC4A4**)	Ion transport, Sodium transport, Symport, Transport
AM869057	Immunoglobulin epsilon Fc receptor Iso2 (**Fcer2**)	Immunity
AM861031	Ubiquitin carboxyl-terminal hydrolase	Immunity, Innate immunity,

	7 (**USP14**)	Ubl conjugation pathway
AM867288	Glutathione S-transferase Y1	Metabolism
AM868209	E3 ubiquitin-protein ligase **MIB2**	Notch signaling pathway, Ubl conjugation pathway
FP011170	Baculoviral IAP repeat-containing protein 7-B (**birc7-b**)	Apoptosis, Ubl conjugation pathway
CU986307	ATP-dependent DNA helicase **PIF1**	DNA damage, DNA recombination, DNA repair
FP003510	Mediator of RNA polymerase II transcription subunit 27 (**MED27**)	Transcription regulation
CU997866	Tumor suppressor candidate 3 (**TUSC3**)	Transport
CU983908	Testis-expressed sequence 33 protein Iso2 (**TEX33**)	Unknown
AM865729	Manganese-dependent ADP-ribose/CDP-alcohol diphosphatase (**ADPRM**)	nucleobase-containing small molecule catabolic process
AM860224	Tripartite motif-containing protein 2 (**TRIM2**)	Ubl conjugation pathway
FP003669	V-type proton ATPase 116 kDa subunit a Iso1 (**ATP6V0A1**)	Ion transport
FP004994	Spermatogenesis-associated protein 7 (**SPATA7**)	Sensory transduction
CU995574	Putative gamma-glutamyltransferase **ywrD**	Glutathione catabolic process
FP009897	1,2-dihydroxy-3-keto-5-methylthiopentene dioxygenase (**Adi1**)	Amino-acid biosynthesis, Methionine biosynthesis
AM854745	Ubiquitin-conjugating enzyme E2 D4 (**UBE2D4**)	Ubl conjugation pathway
CX069279	Carboxypeptidase B (**CPB1**)	Proteolysis
CU683581	Golgi phosphoprotein 3 (**GOLPH3**)	Protein transport
AM855770	Neurogenic locus notch homolog protein 1 (**NOTCH1**)	Angiogenesis, Differentiation, Notch signaling pathway, Development, Transcription regulation
FP007485	Sodium-coupled monocarboxylate transporter 1 (**SLC5A1**)	Apoptosis, Ion transport, Sodium transport, Symport
AM856659	CUB and sushi domain-containing protein 3 (**CSMD3**)	Regulation of dendrite development
CX069305	Complement C1q-like protein 4 (**C1QL4**)	Negative regulation of ERK1 and ERK2 cascade, may inhibit adipocyte differentiation at an early stage
CU999029	Proteasome subunit alpha type-1	Circadian rhythm,

	(**Prosalpha6**)	proteasome-mediated ubiquitin-dependent protein catabolic process, Toll signaling pathway
CU987396	Coiled-coil domain-containing protein 178 (**CCDC178**)	Unknown
CU992173	28S ribosomal protein S12, mitochondrial (**MRPS12**)	Mitochondrial translation
AM861630	Rhomboid-related protein 4 (**RHHBDD1**)	Apoptosis, Differentiation, Spermatogenesis
CU987910	Speckle-type POZ protein (**SPOP**)	Ubl conjugation pathway
CX069297	Neuroendocrine convertase 1 (**PCSK1**)	Cell signaling, peptide and protein processing
CU994588	Ankyrin repeat and SOCS box protein 3 (**ASB3**)	Ubl conjugation pathway
FP001911	Synoviocyte proliferation-associated in collagen-induced arthritis protein 1 (**Saal1**)	Response to stimulus, cell proliferation
CU990223	Integumentary mucin C.1	Reponse to stimulus, immunity
CU685643	Receptor-type tyrosine-protein phosphatase kappa (**PTPRK**)	Cell cycle, cell proliferation, cell migration, transcription
CU988555	Pre-mRNA-splicing factor **SYF2**	Gastrulation, embryonic organ development, cell cycle, RNA splicing
CU991046	Homeobox protein orthopedia (**Otp**)	Differentiation, Neurogenesis, Transcription regulation
AM854715	Fatty acyl-CoA hydrolase precursor, medium chain	Fatty acid biosynthesis, Fatty acid metabolism, Lipid biosynthesis, Lipid metabolism
FP007109	EAL domain-containing protein	Unknown
